# Semi‐parametric Regression under Model Uncertainty: Economic Applications[Link]

**DOI:** 10.1111/obes.12294

**Published:** 2019-02-19

**Authors:** Gertraud Malsiner‐Walli, Paul Hofmarcher, Bettina Grün

**Affiliations:** ^1^ Department of Finance, Accounting and Statistics Vienna University of Economics and Business (WU) Welthandelsplatz 1 1020 Vienna Austria; ^2^ Salzburg Centre of European Union Studies (SCEUS) Department of Business, Economics and Social Theory Paris Lodron University of Salzburg Mönchsberg 2a 5020 Salzburg Austria; ^3^ Department of Applied Statistics Johannes Kepler University Linz Altenbergerstraße 69 4040 Linz Austria

## Abstract

Economic theory does not always specify the functional relationship between dependent and explanatory variables, or even isolate a particular set of covariates. This means that model uncertainty is pervasive in empirical economics. In this paper, we indicate how Bayesian semi‐parametric regression methods in combination with stochastic search variable selection can be used to address two model uncertainties simultaneously: (i) the uncertainty with respect to the variables which should be included in the model and (ii) the uncertainty with respect to the functional form of their effects. The presented approach enables the simultaneous identification of robust linear and nonlinear effects. The additional insights gained are illustrated on applications in empirical economics, namely willingness to pay for housing, and cross‐country growth regression.

## 1 Introduction

Model uncertainty is pervasive in empirical economics. Economic theory does not always specify or is inconclusive with respect to (i) the covariates which are part of the model, and (ii) the functional form between dependent and explanatory variables. Bayesian model averaging (henceforth BMA) has become a popular tool to perform robust inference under model uncertainty (Hofmarcher *et al*., 2018). The model uncertainty usually accounted for in the BMA framework only relates to variable uncertainty while assuming that the relationship between dependent and explanatory variables is linear. For instance, in the field of long‐term *per capita* GDP growth empirics a huge strand of literature applies BMA methods to identify robust linear determinants for growth. Variable uncertainty arises in this context due to the so‐called *Open‐endedness* of theory (see Brock and Durlauf, 2001) where competing growth theories do not rule out each other. BMA takes this variable uncertainty into account (see, e.g. Fernández, Ley and Steel, 2001; Sala‐i‐Martin, Doppelhofer and Miller, 2004; Amini and Parmeter, 2012; Ley and Steel, 2012). BMA techniques are also employed in Mitchell, Pain and Riley (2011) to identify economic and policy drivers of international migration to the UK, in Eicher, Henn and Papageorgiou (2012) to assess effects of preferential trade agreements on trade flows, and in Tobias and Li (2004) to determine returns to education.

Although the insights from these BMA exercises are valuable, they rely on the assumption that the functional relationship between the potential covariates and the dependent variable is known. In standard BMA all covariates included in the model are assumed to have a linear effect on the outcome. A nonlinear functional relationship is modelled by manually creating and adding new covariates, e.g. by including polynomial terms (Henderson and Parmeter, 2016) or defining a piecewise linear function (Tobias and Li, 2004). Special care is needed if these additional terms are to be included in the model in a hierarchical or grouped way. For instance, if interaction effects are only to be included jointly with their main effects, the constraint has to be imposed that main effects need to be included first before interaction effects can be included (Crespo Cuaresma, 2010; Montgomery and Nyhan, 2010). The inclusion of grouped terms representing a nonlinear effect requires that all terms needed to model nonlinearity are either jointly added or dropped from the model. Due to this manual tuning required for the inclusion of any nonlinear effect, BMA applications in general only include a small subset of potential covariates with nonlinear effects.

However, functional misspecification occurs if covariates are only included with linear effects while in fact their effects are actually nonlinear. In this case, several issues may arise which preclude the correct robust identification of effects: (i) irrelevant covariates may be included into the model to compensate for the missing nonlinear effects and are overestimated in terms of importance, (ii) important nonlinear covariates may not be identified due to the functional misspecification, and (iii) effects of covariates, which vary over the covariate range, are forced to be constant over the entire covariate range. Inference based on an improperly specified model thus may lead to incorrect conclusions and policy prescriptions.

Therefore, efforts have been made in empirical economics to develop and apply modelling techniques which uncover also nonlinear effects without the need to know and a priori explicitly define these nonlinear effects. Non‐parametric as well as semi‐parametric methods have been suggested. Non‐parametric methods used in economic regression applications are mainly based on kernel estimation (for an overview see Henderson and Parmeter, 2015). For instance, local‐linear least square regressions are used in Henderson, Papageorgiou and Parmeter (2011) to investigate nonlinearities of different economic growth determinants and in Delgado, Henderson and Parmeter (2014) to study the effect of education on economic growth. Semi‐parametric methods using model matrix expansions to obtain more flexible regression functions are discussed in Smith and Kohn (1996) to model housing values, and in Koop and Tobias (2006) to study returns to education.

Compared to BMA, where dozens of potential determinants are simultaneously included in the analysis to identify the relevant ones, the non‐ and semi‐parametric methods considered so far were only used with a limited subset of covariates. Henderson *et al*. (2011) consider at most 11, Smith and Kohn (1996) five and Henderson and Parmeter (2016) two nonlinear regressors. Thus these approaches account for functional uncertainty, but only after having restricted the potential set of covariates and relying on prior knowledge to reduce variable inclusion uncertainty. The results obtained using these approaches therefore do not account for the complete model uncertainty. This shortcoming is also pointed out by Henderson *et al*. (2011), who state that future research should focus on merging the use of nonlinear regression methods with BMA techniques. The approach presented in this paper addresses this issue. In particular, we propose to use recently developed Bayesian variable selection methods which incorporate nonlinear effect estimation into the BMA framework using a semi‐parametric approach (Scheipl, Fahrmeir and Kneib, 2012). In the following, we refer to this approach as Spike‐Slab‐GAM (henceforth, SSG).

The SSG approach is appealing for several reasons. In contrast to standard BMA, SSG simultaneously accounts for two sources of model uncertainty, the variable inclusion uncertainty and the functional form uncertainty of each variable. SSG estimates the functional relationships between the covariates and the dependent variable in a very flexible way through smooth functions, where their specific form does not need to be a priori specified. For each covariate, two terms are included, a linear and a nonlinear term. Each of these two terms can be selected independently and the inclusion probability of both terms can be evaluated separately. The results indicate for each covariate (i) if it is a robust linear determinant of the outcome and (ii) if it has robust nonlinear effect on the outcome.

The smooth functions are modelled using penalized spline regression (Eilers and Marx, 1996). This alleviates the problem of specifying an appropriate spline basis by selecting a suitable order, number and location of the basis functions, and gives good results as long as a flexible enough spline basis is specified. A specifically designed prior construction on the regression coefficients regularizes the estimation of the high‐dimensional parameter vector and allows simultaneous consideration of a large number of nonlinear effects. For example, when analysing the data set by Sala‐i‐Martin *et al*. (2004) in section 5 more than 50 nonlinear effects of covariates are simultaneously considered.

The results of SSG can be analysed and interpreted in a similar way to those obtained from standard BMA. The same key characteristics, such as posterior inclusion probabilities, are available for each linear and nonlinear term to assess the effect of a covariate. Thus, SSG identifies the robust linear and nonlinear effect of a covariate and indicates whether a covariate is a relevant linear and/or nonlinear driver of the outcome or not relevant at all.

The SSG model specification can also be modified in the same ways as suggested previously for BMA. For example, as a response to Ciccone and Jarociński (2010) who criticize the instability of BMA results, Rockey and Temple (2016) suggest to pursue a less ‘agnostic’ approach and to restrict the model space by forcing some covariates to be included. This idea can also be used with SSG to selectively include covariates with specific functional relationships. In a similar vein, also interaction terms as considered by Montgomery and Nyhan (2010), can be added or the inclusion of bivariate nonlinear terms considered.

Spike‐Slab‐GAM differs from standard BMA employed in empirical economics in two ways: (i) nonlinear effects are specified with an a priori unknown functional form, and (ii) a slightly different prior on the regression coefficients is imposed to perform variable selection. The modification of the prior structure is necessary to include or exclude all coefficients belonging to the same nonlinear effect jointly and to simultaneously estimate inclusion probabilities and the smoothness penalty in a computationally feasible way.

Our work is organized as follows: In section 2, we present the details of model specification for SSG. We indicate how SSG identifies robust determinants under variable uncertainty while supporting arbitrary functional nonlinear relationships between the covariates and the outcome. More details on the methodology are provided in online Appendix B where a general statistical framework is presented and discussed which contains the BMA and SSG approaches as special cases. The importance of accounting for nonlinear effects is illustrated in a simulation study in section 3, which provides a comparison between BMA using a standard implementation (Zeugner and Feldkircher, 2015) and SSG. We illustrate that SSG is superior to BMA if nonlinearities are present in the data. The simulation study also indicates that the cost associated with using SSG compared to BMA is negligible, even if the true functional relationship is linear. SSG is applied to economic applications consisting of willingness to pay for housing (Harrison Jr. and Rubinfeld, 1978) in section 4, and to cross‐country growth regression (Fernández *et al*., 2001; Sala‐i‐Martin *et al*., 2004) in section 5. In these applications, well‐known data sets are reanalysed using SSG, and results are compared to BMA and a variant of SSG where only linear effects are included. The effect estimates of the covariates exhibiting nonlinear effects are visualized. The insights gained by including the nonlinear effects are pointed out. Finally, section 6 concludes.

## 2 Methodology

This section describes the model specification of SSG as proposed by Scheipl *et al*. (2012) and discusses estimation, inference and postprocessing tools. First, linear additive models based on penalized splines are introduced. This flexible model class is able to fit any smooth function to represent the functional relationship between a covariate and the outcome variable. Then, the linear additive model is combined with stochastic search variable selection. This combination accounts for variable inclusion and functional relationship uncertainty. Finally, suitable posterior inference tools to assess a covariate's importance and its robust effect are discussed.

### Linear additive models with penalized splines

We assume the following regression setting of *n* observations with a dependent variable $y\in R^{n}$ and a set of explanatory variables $x_{k}\in R^{n}$, *k*=1,…,*K*, where the dependent variable is assumed to be given by

$$y=\alpha \iota +\sum_{k=1}^{K}\;f_{k}\left(x_{k}\right)+\epsilon ,$$

with ***ι*** a vector of ones of length *n*,* α* the intercept, the function *f*_*k*_(·) capturing the influence of each covariate ***x***_*k*_ on the dependent variable and ***ε*** a length *n* vector of i.i.d. normally distributed noise with mean 0 and variance *σ* ^2^. This kind of regression model is also referred to as a ‘linear additive model’ (Wood, 2006).

The unknown functions *f*_*k*_(·) are assumed to have mean zero (for identifiability of the intercept) and to be smooth, but are otherwise not further specified or restricted. Different modelling approaches have been proposed to estimate *f*_*k*_(·) including non‐parametric approaches (Li and Racine, 2007) and semi‐parametric methods based on splines, see, e.g. Smith and Kohn (1996) and Wood (2006).

Splines are smooth functions which approximate any kind of functional shape in a flexible way. They are used in a regression framework in case the functional form of the effects is unknown. Splines are defined by piecewise polynomials which are generated by basis functions. The spline basis expansion leads to an extended model matrix for the regression model. Using such a spline basis expansion in the regression implies that the smooth function is approximated by

(1) $$f_{k}\left(x_{k}\right)\approx S_{k}{\tilde{\delta }}_{k},$$

with $S_{k}\in R^{n\times (L_{k}+1)}$ the spline basis expansion of ***x***_*k*_ with dimension *L*_*k*_+1 and ${\tilde{\delta }}_{k}$ the regression coefficients.

The selection of the order of the polynomials, the number of knots acting as anchor points for the polynomial pieces and the location of the knots are crucial choices for the spline basis. All these parameters determine how flexible the spline function is. If the polynomial order or the number of knots is too low or the locations are not well selected the functional relationship between covariate and dependent variable cannot be estimated well, but a bias is introduced. On the other hand, if the polynomial order and the number of locations are too high, too wiggly and overfitting curves may be obtained.

For penalized splines these specifications are less crucial. Penalized splines are used in combination with a generous spline basis expansion which includes more flexibility than actually needed. The appropriate smoothness of the estimated function is then determined by imposing a penalty on the regression coefficients of the spline basis expansion. Thus, the problem of selecting the polynomial order and the number and location of the knots is reduced to the problem of selecting a suitable penalty value. This considerably facilitates the model specification because default settings can be employed for the basis expansion (Wood, 2006).

Eilers and Marx (1996) propose to use penalized cubic *B*‐splines as basis functions. They exploit that *B*‐splines only act locally, i.e. they are non‐zero only on a sub‐interval of the interval where the function is defined, and impose a penalty on the second differences of the coefficients of adjacent *B*‐splines to induce smoothness of the estimated curve. Using the second differences as a penalty implies that constant and linear functions do not incur a penalty, but other local changes in functional shape are penalized. The regression coefficients *α* and ${\tilde{\delta }}_{k}$, *k*=1,…,*K*, are then determined by minimizing the ordinary least squares (OLS) criterion plus the penalty term, i.e.

(2) $${\left(y-\alpha \iota -\sum_{k=1}^{K}S_{k}{\tilde{\delta }}_{k}\right)}^{⊤}\left(y-\alpha \iota -\sum_{k=1}^{K}S_{k}{\tilde{\delta }}_{k}\right)+\sum_{k=1}^{K}\lambda_{k}{\left(\Delta^{2}{\tilde{\delta }}_{k}\right)}^{⊤}\left(\Delta^{2}{\tilde{\delta }}_{k}\right),$$

where $\Delta^{2}{\tilde{\delta }}_{k}$ denotes the second differences of vector ${\tilde{\delta }}_{k}$. Suitable values for the penalty parameter *λ*_*k*_, *k*=1,…,*K* need to be determined such that a smooth function is obtained without overfitting the data. For *λ*_*k*_=0 the fitted model corresponds to the OLS fit given the spline basis expanded model matrix of ***x***_*k*_, while for *λ*_*k*_ tending to ∞ the OLS fit for the model matrix including only the intercept and the linear term ***x***_*k*_ is obtained, i.e. the fitted smooth function is shrunken towards the best‐fitting linear function.

The penalized estimation (2) corresponds to a Bayesian regression analysis where a normal prior on the regression coefficients is imposed (Lang and Brezger, 2004; Fahrmeir, Kneib and Konrath, 2010). The penalty parameters *λ*_*k*_, *k*=1,…,*K*, are related to the precision parameters in these priors and a hierarchical prior enables a data‐driven estimation of the smoothness penalty.

Since second‐order differences lead to shrinkage towards a linear function, the constant and linear terms are not penalized in this setting. Therefore, in order to ensure a proper prior structure Scheipl *et al*. (2012) propose to split the smooth function given in equation (1) into two components: (i) the linear term and (ii) the nonlinear term corresponding to the part of the smooth function which is orthogonal to intercept and linear term, i.e. with mean and slope zero. The model matrix ***S***_*k*_ is thus split into two model matrices. One matrix consists of the linear term ***x***_*k*_, the other matrix ***Z***_*k*_ is the orthogonal complement to the intercept and linear term, capturing the deviation of *f*_*k*_(·) from the linear function:

$$S_{k}{\tilde{\delta }}_{k}=x_{k}\beta_{k}+Z_{k}\delta_{k},$$

where $Z_{k}\in R^{n\times L_{k}}$.

Using this representation, the final regression model is given by

(3) $$y=\alpha \iota +\sum_{k=1}^{K}\left(x_{k}\beta_{k}+Z_{k}\delta_{k}\right)+\epsilon ,$$

where ***δ***_*k*_ needs to be suitably penalized to obtain a function with appropriate smoothness.

### Stochastic search variable selection

The aim of SSG is to infer if ***x***_*k*_ has a linear, a nonlinear, or a smooth effect (which results from a combination of linear and nonlinear effect) on the outcome. This can be determined by estimating whether the coefficients *β*_*k*_, ***δ***_*k*_, or both, are different from zero. In a Bayesian variable selection setting, priors for *β*_*k*_ and ***δ***_*k*_ are specified to enable this identification. Variable selection priors considered in the Bayesian literature so far can be roughly classified into two groups, shrinkage priors and spike‐and‐slab priors.

Shrinkage priors are prior distributions with a lot of mass at and/or close to zero. This reflects the belief that the coefficients may eventually be zero and the variables omitted from the model. Examples for shrinkage priors are the Laplace prior, which corresponds to lasso estimation in a frequentist setting (Tibshirani, 1996; Park and Casella, 2008), or more recently, the Dirichlet–Laplace prior (Bhattacharya *et al*., 2015).

In contrast, spike‐and‐slab priors (Mitchell and Beauchamp, 1988) impose a two‐component mixture distribution on the coefficients *β*_*k*_ and ***δ***_*k*_ for *k*=1,…,*K*. Both mixture components have mean zero, however, the variances differ. One component has all its mass close to zero or is even a degenerate point mass distribution at zero, called the ‘spike’, and the other component follows a flat distribution which would be used as prior if it were clear that this variable should be included into the model (the ‘slab’). For an overview on spike‐and‐slab priors see, for example, O’Hara and Sillanpää (2009) and Malsiner‐Walli and Wagner (2011).

Both approaches, the standard BMA as well as the proposed SSG, implement special cases of a spike‐and‐slab prior. Compared to shrinkage priors, spike‐and‐slab priors have the advantage that assessment of effect importance is straightforward and can be based on inference tools developed for BMA. For instance, the importance of a regressor is determined by evaluating the posterior weight of slab assignment for this regressor, the so‐called ‘posterior inclusion probability’.

In standard BMA the spike‐and‐slab prior has a spike distribution which consists of a degenerate distribution with point mass at zero and a slab distribution based on the normal *g*‐prior (see, e.g. Zeugner and Feldkircher, 2015). In SSG, the spike‐and‐slab prior consists of a ‘parameter‐expanded normal‐mixture of inverse Gammas (peNMIG) prior’. This prior builds on the normal‐mixture of inverse Gammas (NMIG) prior proposed by Ishwaran and Rao (2005). The prior is denoted by the following:

$$\beta_{k},\delta_{k}∼\text{peNMIG}\left(v_{0},w,a_{\tau },b_{\tau }\right),\;k=1,\ldots ,K,$$

where *a*_*τ*_ and *b*_*τ*_ are the parameters of the inverse Gamma prior for the slab variance, *ν*_0_ is the ratio between spike and slab variance and (*w*,1−*w*) are the weights of the two‐component mixture. Additionally, the weight *w* is assumed to follow a Beta distribution:

(4) w∼Beta(a,b).

The peNMIG prior enables the joint inclusion and exclusion of coefficients of the linear terms *β*_*k*_ and the nonlinear terms ***δ***_*k*_ together with the determination of a suitable penalty, i.e. smoothness, for the nonlinear function.^1^

In addition to the priors on the regression coefficients which enable stochastic search variable selection, priors for the intercept *α* and the error variance *σ* ^2^ are also needed for Bayesian inference. A standard specification is used assuming

$$\alpha ,\sigma^{2}\propto {\text{Gamma}}^{-1}\left(a_{\sigma },b_{\sigma }\right).$$

Markov chain Monte Carlo (MCMC) methods are employed to perform Bayesian inference for SSG. Analogous to standard BMA, data augmentation is performed and a term inclusion indicator vector ***γ*** is introduced which is added as a latent variable to the sampling scheme. The binary indicator vector ***γ*** takes $2^{2K}$ different values in {0,1}^2*K*^ and indicates for each term whether it is assigned to the slab or the spike. In standard BMA, a collapsed Gibbs sampling scheme is used where only ***γ*** is sampled, whereas all other parameters are integrated out. By contrast, a block‐wise Gibbs sampler is used for estimating SSG where the regression coefficients are not integrated out but are also sampled in addition to the term inclusion vector ***γ***. A detailed description of the MCMC methods for estimating SSG are outlined in Scheipl (2011) and Scheipl *et al*. (2012).

### Posterior inference

The term inclusion indicator vector ***γ*** indicates whether a coefficient is assigned to the slab or to the spike component. Assignment to the spike is interpreted as having the coefficient excluded from the model, while assignment to the slab implies that the same prior is imposed on the regression coefficient as if the covariate had been a priori included. Therefore, the indicator variable ***γ*** is used to determine the importance of a linear or nonlinear term through the posterior inclusion probability (PIP), i.e., the posterior expectation of the term being assigned to the slab. Each ***γ*** can be interpreted as a different model *M*. The posterior inclusion probability for term *k*, where *k*=1,…,2*K* comprising linear and nonlinear terms, is then given by

(5) $${\text{PIP}}_{k}=\sum_{j=1}^{2^{2K}}1\left(\gamma_{k}=1|M_{j}\right)P\left(M_{j}|y\right).$$

and is estimated by the proportion of MCMC samples for which *γ*_*k*_=1.

The posterior mean (PM) for the linear term of variable *k* is determined by

$${\text{PM}}_{k}=\sum_{j=1}^{2^{2K}}P\left(\beta_{k}|M_{j}\right)P\left(M_{j}|y\right),$$

and the conditional positive sign certainty (PSC) for the linear term of variable *k* by

$${\text{PSC}}_{k}=\frac{\sum_{j=1}^{2^{2K}}P\left(\beta_{k}>0|M_{j}\right)P\left(M_{j}|y\right)}{\sum_{j=1}^{2^{2K}}1\left(\gamma_{k}=1|M_{j}\right)P\left(M_{j}|y\right)}.$$

While PIP is a measure of variable importance, PM indicates the robust estimate of the effect of the regressor across all sampled models, and PSC is a measure for the posterior confidence in the sign of this robust effect estimate being positive (Sala‐i‐Martin *et al*., 2004).

For hierarchical Bayesian models, the deviance information criterion (DIC) has been suggested as a general criterion to assess and compare the goodness‐of‐fit of different models (Spiegelhalter *et al*., 2002). The DIC can be directly calculated from the MCMC draws and is given by the posterior mean deviance plus the effective number of parameters. The effective number of parameters is estimated by the difference between the posterior mean deviance and the deviance at the posterior mean. Smaller values of the DIC indicate a better model fit.^2^

## 3 Simulation study

In the simulation study, it is investigated whether (i) BMA and SSG perform similarly well if the assumption that the covariates affect the dependent variable linearly holds and (ii) SSG provides suitable results in case this assumption is violated and a nonlinear effect is present whereas BMA fails to identify the relevant covariates.

### Design and specifications

Two different simulation settings are considered depending on whether only linear effects (‘linear setting’) or linear effects and an additional nonlinear effect determine the outcome (‘nonlinear setting’). For each setting 100 data sets are generated with each data set consisting of 50 observations and 35 covariates. In both settings five of the covariates have a linear effect. In the nonlinear setting an additional variable with a nonlinear effect is included. In particular, for the linear setting the dependent variable is given by

$$y_{i}=\sum_{k=1}^{5}x_{\mathrm{ik}}+\epsilon_{i},$$

while in the setting containing a nonlinear effect the dependent variable is given by

$$y_{i}=\sum_{k=1}^{5}x_{\mathrm{ik}}-1.5x_{i6}^{2}+\epsilon_{i}.$$

The remaining covariates have no effect on the dependent variable. All covariates are drawn from a multivariate normal distribution with mean zero and variance one and with either (i) zero correlations or (ii) correlations equal to 0.5. The errors *ε*_*i*_ are drawn from *N*(0,1).

The simulation study compares the results for BMA and SSG where for SSG two model specifications are considered: (i) in “SSG‐linear” only linear terms and (ii) in “SSG‐smooth” linear and nonlinear terms are included. For BMA 1,000,000 iterations are performed after a burn‐in of 20,000 iterations; for SSG 35,000 iterations after a burn‐in of 5,000 iterations are made. Model fitting is performed using standardized covariates and dependent variable.^3^

For each data set the results are evaluated by determining (i) the mean posterior model size, (ii) the number of true positives, i.e., the number of covariates among all selected covariates which truly have an effect on the outcome, (iii) the mean absolute deviation (MAD) between the true inclusion indicator and the estimated posterior inclusion probability, (iv) the mean squared error (MSE) between the true and predicted outcome, and (v) the DIC values. Aggregate results over the 100 data sets are reported by determining the median and the robust standard deviation.

The mean posterior model size is estimated by the average number of covariates which are assigned to the slab distribution. The number of true positives TP_*j*_ for model *M*_*j*_ corresponds to the number of covariates which are contained in the true model as well as in *M*_*j*_ (as induced by the assignments to the slab). More formally, TP_*j*_ is determined by

$${\text{TP}}_{j}=\sum_{v=1}^{V}\left(1-\prod_{k\in K_{v}}\left(1-1\left(\gamma_{k}=1|M_{j}\right)\right)\right),$$

where *V* is the number of variables with non‐zero effects (*V*=5 in the linear setting and *V*=6 in the nonlinear setting) and *K*_*v*_ is the set of terms associated with variable *v*. For BMA and SSG‐linear *K*_*v*_ contains a single term, while for SSG‐smooth *K*_*v*_ contains two terms, the linear and the nonlinear term associated with variable *v*. To determine the overall TP criterion for one data set the median number of true positives is estimated based on all models, weighted with their posterior evidence.

The MAD criterion for variable inclusion is estimated by

$${\text{MAD}}_{\mathrm{PIP}}=\frac{1}{K}\sum_{k=1}^{K}\left\|{\hat{\text{PIP}}}_{k}-{\text{PIP}}^{\text{true}}\right\|,$$

with ${\hat{\text{PIP}}}_{k}$ the estimated PIP of variable *k* and *K*=35 in this simulation study. In the nonlinear setting, ${\hat{\text{PIP}}}_{k}$ corresponds to the estimated posterior probability of including either a linear and/or a nonlinear term.

The MSE criterion to assess predictive performance is estimated by

$${\text{MSE}}_{y}=\frac{1}{n}\sum_{i=1}^{n}{\left({\hat{y}}_{i}-y_{i}\right)}^{2},$$

with ${\hat{y}}_{i}$ the predicted outcome and *y*_*i*_ a newly sampled outcome value given the same covariates ***x***_*i*_ in order to avoid an in‐sample bias. Predictions are obtained based on posterior mean estimates of the regression coefficients.

### Results

Simulation outcomes are summarized in Table 1. The upper part of the table provides the results for the setting where only linear effects affect the outcome. In this case all three approaches are able to identify the correct model. The estimated models contain all relevant variables regardless of if the covariates are independent or correlated (median TP = 5) and only a small number of unrelated covariates are additionally included, as indicated by the median model sizes which are between 6.4 and 8.2. According to the MAD criterion the expected probability of wrongly excluding a relevant variable or wrongly including an irrelevant one is small for all three approaches. With respect to MSE BMA and SSG‐linear seem to perform slightly better than SSG‐smooth. This may be due to the additional complexity involved when fitting SSG‐smooth. The DIC values suggest that all three approaches result in a similar model fit.

**Table 1:** Simulation results

*Setting*	*Estimation method*	*Model size*	*TP*	*MAD*_PIP_	*MSE*_*y*_	*DIC*
Linear, uncorrelated	BMA	7.8 (0.9)	5 (0)	0.08 (0.02)	0.21 (0.04)	71.0 (11.6)
	SSG‐linear	6.4 (0.5)	5 (0)	0.04 (0.01)	0.21 (0.04)	73.6 (10.2)
	SSG‐smooth	8.1 (0.7)	5 (0)	0.08 (0.02)	0.23 (0.05)	74.8 (11.1)
Linear, correlated	BMA	7.6 (1.1)	5 (0)	0.08 (0.03)	0.23 (0.05)	71.1 (11.3)
	SSG‐linear	6.4 (0.4)	5 (0)	0.04 (0.01)	0.23 (0.05)	74.5 (9.9)
	SSG‐smooth	8.2 (0.7)	5 (0)	0.08 (0.02)	0.25 (0.05)	74.9 (10.8)
Nonlinear, uncorrelated	BMA	2.8 (0.9)	1 (1)	0.16 (0.01)	0.63 (0.09)	140.7 (4.1)
	SSG‐linear	3.4 (0.6)	2 (0)	0.15 (0.01)	0.56 (0.05)	134.1 (4.6)
	SSG‐smooth	9.2 (0.5)	6 (0)	0.06 (0.01)	0.12 (0.02)	50.9 (11.3)
Nonlinear, correlated	BMA	6.2 (2.6)	4 (1)	0.14 (0.01)	0.37 (0.11)	119.2 (15.1)
	SSG‐linear	5.3 (1.2)	4 (1)	0.11 (0.02)	0.39 (0.09)	118.9 (10.2)
	SSG‐smooth	8.6 (0.7)	6 (0)	0.06 (0.01)	0.15 (0.03)	56.3 (10.5)

*Notes*: Median values with robust standard deviations in parentheses over 100 data sets are given for each criterion. The correct number of true positives (TP) is five in the linear and six in the nonlinear setting.

The lower part of Table 1 provides the results for the nonlinear setting. In this case BMA as well as SSG‐linear misspecify the model, while SSG‐smooth is able to identify the nonlinear effect and encompasses the correct model specification. Clearly BMA and SSG‐linear perform worse than SSG‐smooth regardless of the correlation structure between the covariates. In the case of uncorrelated covariates the estimated models are too small (with a median model size of 2.8 and 3.4) and hardly contain any relevant covariates (median TP = 1 and 2). This issue is less pronounced in the case of correlated covariates. However, still not all relevant covariates are included, but some irrelevant ones are contained. In the case of correlated covariates also a high variability in the mean posterior model size is observed, as indicated by the robust standard deviations which are 2.6 for BMA and 1.2 for SSG‐linear. This inferior performance is also reflected by the higher values for MAD_PIP_ and MSE_*y*_ compared to SSG‐smooth. SSG‐smooth identifies all relevant covariates and also provides good PIP estimates and predictive performance. The DIC values indicate that SSG‐smooth provides a considerably better model fit than BMA and SSG‐linear.

**Figure 1:**
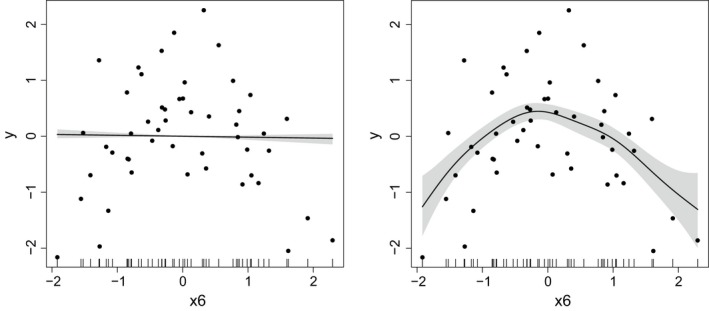
Simulation results, nonlinear data set, uncorrelated covariates: Estimated effect of variable *x*_6_ using SSG‐linear (on the left) or SSG‐smooth (on the right).

For one nonlinear uncorrelated data set, Figure 1 shows the estimated effect of covariate *x*_6_, if SSG‐linear (on the left) and SSG‐smooth (on the right) are used for model fitting. The posterior medians of the smooth effects are indicated by the black lines as well as the 80% confidence bands indicated by the grey shaded areas. Clearly for SSG‐linear, where the effect is restricted to be linear, no impact of *x*_6_ on the dependent variable is detected, while the nonlinear effect is identified when using SSG‐smooth.

In the simulation study BMA and SSG‐linear give similar results despite implementing a different variant of a spike‐and‐slab prior and imposing different priors on the regression coefficients. Furthermore, SSG‐smooth provides a suitable approach to identify robust determinants and achieve good predictive performance when a nonlinear relationship between the covariates and the dependent variable cannot be safely a priori excluded. Also, the cost of switching to this approach is small if the linear model constitutes the correct specification. The use of SSG‐smooth safeguards against obtaining completely unreliable results if the assumption of a linear relationship is violated.

## 4 Application to the Boston housing data set

Smith and Kohn (1996) used the Boston housing data set to indicate the advantages of selectively including nonlinear terms when predicting median home values from geographical and infrastructure characteristics of the property while accounting for model uncertainty. Thus, the aim in the following is to replicate the nonlinear effects reported by Smith and Kohn (1996) in an analysis where all variables are considered to have effects that are potentially nonlinear.

Results are presented for BMA, SSG‐linear and SSG‐smooth. This enables a comparison between BMA and SSG‐linear to assess the impact of the different prior constructions. The results for SSG‐smooth then provide insights into the differences obtained due to the additional inclusion of nonlinear effects. The results also indicate the usefulness of SSG compared to BMA in detecting nonlinear relationships.

### Design and specifications

Before analysis the data set was standardized. This ensures that a similar amount of shrinkage in terms of standard deviations of the covariates is imposed on each of the covariates. BMA was run with 2,000,000 burn‐in iterations and 2,000,000 recorded iterations, while SSG was run with 10 parallel chains using 2,000,000 burn‐in iterations and 200,000 iterations recorded with a thinning of 5 for each chain. The same estimation methods and hyper‐parameter values are used as in the simulation study in section 3.^4^

The results for BMA, SSG‐linear and SSG‐smooth are assessed by a detailed comparison of the estimated PIPs, PMs and PSCs for the linear terms and the estimated PIPs for the nonlinear terms. In addition the posterior model size distributions of SSG‐linear and SSG‐smooth are compared where the model size is determined by the number of included linear and nonlinear terms. The posterior model size distribution is further inspected for SSG‐smooth by splitting the number of included terms into the number of linear terms, the number of nonlinear terms without the linear term of this variable included and the number of nonlinear terms with the linear term of this variable included. For the variables with the highest posterior evidence for a nonlinear effect the smooth effects, i.e. the combined linear and nonlinear effects of a covariate, are visualized by their 80% confidence bands using the corresponding 10% and 90% quantiles of the posterior distributions as well as by the median of the posterior distributions.

### Results

Harrison Jr. and Rubinfeld (1978) investigated the willingness to pay for clean air using data for the Boston metropolitan area. This Boston housing data set was also used by Smith and Kohn (1996) to present their approach where the functional relationship between a small subset of selected covariates and the dependent variable was specified as potentially nonlinear. The data set consists of 506 observations on 13 covariates to predict the median price of owner‐occupied homes in USD 1000's based on data from the 1970 census.^5^ In contrast to Smith and Kohn (1996), where only five covariates are modelled nonlinearly, in SSG‐smooth all covariates (except for the Charles river dummy) are included with linear and nonlinear effects.

Table 2 reports the results for BMA, SSG‐linear and SSG‐smooth. For the Charles River dummy (chas) no nonlinear effect is estimated. This is indicated in the column PIP for the nonlinear effects by a dash (–). The results for BMA and SSG‐linear are very similar in terms of PIP, PM and PSC. Both identify nearly all covariates as being robust determinants for the willingness to pay for housing. Only proportion of non‐retail business acres per town (indus) and the proportion of owner‐occupied units build prior to 1940 (age) have comparably small PIPs. If SSG‐smooth is used and nonlinear terms are also included in the model, the set of variables identified as robust determinants is considerably reduced. In particular the variables proportion of residential land zoned for lots over 25,000 sq.ft. (zn), some functional expression of the proportion of blacks by town (b) and the Charles River dummy (chas) are consistently not included in the model. These variables would have thus been falsely identified as robust determinants for the willingness to pay for housing when using BMA or SSG‐linear.

**Table 2:** Boston housing data set analysed using BMA, SSG‐linear and SSG‐smooth

	*BMA*			*SSG‐linear*			*SSG‐smooth*			
*Terms*	*Linear*			*Linear*			*Linear*			*Nonlin*.
*Variable*	*PIP*	*PM*	*PSC*	*PIP*	*PM*	*PSC*	*PIP*	*PM*	*PSC*	*PIP*
dis	1.00	−0.34	0.00	0.99	−0.33	0.00	0.76	−0.15	0.00	0.78
lstat	1.00	−0.41	0.00	1.00	−0.41	0.00	1.00	−0.47	0.00	0.97
ptratio	1.00	−0.22	0.00	1.00	−0.22	0.00	0.97	−0.16	0.00	0.13
rm	1.00	0.29	1.00	1.00	0.30	1.00	1.00	0.27	1.00	1.00
nox	1.00	−0.22	0.00	0.97	−0.21	0.00	0.66	−0.13	0.01	0.80
rad	1.00	0.28	1.00	0.94	0.23	1.00	0.77	0.22	1.00	0.12
b	0.99	0.09	1.00	0.81	0.08	1.00	0.15	0.01	0.98	0.09
tax	0.98	−0.21	0.00	0.80	−0.16	0.01	0.52	−0.11	0.04	0.65
zn	0.98	0.11	1.00	0.89	0.10	1.00	0.05	0.00	0.77	0.07
crim	0.98	−0.10	0.00	0.85	−0.08	0.00	1.00	−0.20	0.00	0.39
chas	0.97	0.07	1.00	0.67	0.05	1.00	0.22	0.01	1.00	–
indus	0.25	0.00	0.97	0.11	−0.00	0.51	0.09	−0.01	0.12	0.16
age	0.24	0.00	0.98	0.11	0.00	0.50	0.05	−0.00	0.39	0.07
DIC	782.20			883.81			725.21			

*Notes*: nonlin: nonlinear; PIP: posterior inclusion probability; PM: posterior mean; PSC: posterior conditional positive sign certainty; DIC: deviance information criterion.

The SSG‐smooth results further indicate that the two variables capturing the pupil‐teacher ratio by town (ptratio) and the index of accessibility to radial highways (rad) only have a linear effect on the dependent variable. The covariates describing the average number of rooms per dwelling (rm), percentage of lower status of the population (lstat) and nitric oxides concentration (parts per 10 million; nox) are identified as having a linear effect with an additional nonlinear effect.

The DIC values indicate that SSG‐smooth fits best, followed by BMA and SSG‐linear. The DIC value for SSG‐linear is considerably higher than for BMA. This might be due to the more complex model specification in SSG compared to BMA.

**Figure 2:**
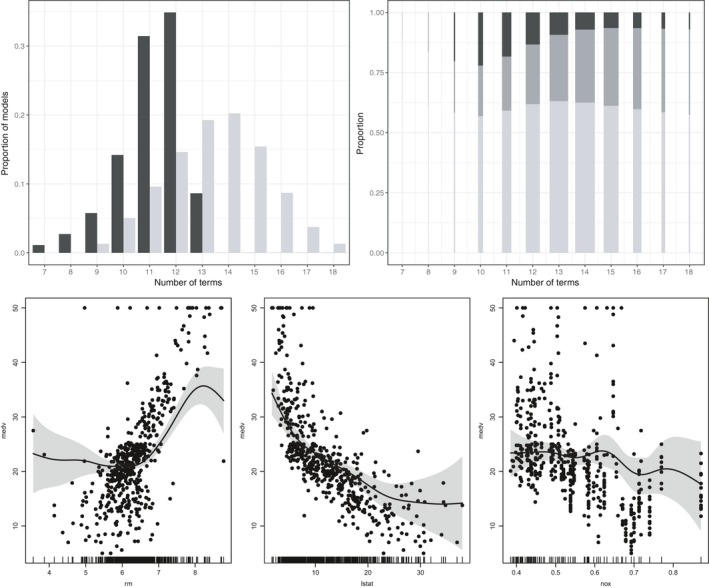
Boston housing data set. Top left: Distribution of model sizes for SSG‐linear (black) and SSG‐smooth (light grey). Top right: Distribution of linear terms (light grey), nonlinear terms with corresponding linear terms included (dark grey) and nonlinear terms without corresponding linear terms (black) given model size for SSG‐smooth. The bar widths are proportional to the posterior probability of the model size. Bottom: Estimated robust smooth effects indicated by the 80% confidence bands and the median of the posterior distribution.

SSG‐linear and SSG‐smooth are also compared with respect to their posterior model size distributions. SSG‐linear leads to an average model size of 11.1, whereas the additional inclusion of nonlinear terms leads to a slightly higher model size of 13.5. The corresponding visualization of the model size distributions is given in Figure 2 on the top left. For SSG‐linear the maximum number of included terms is 13 and most of the models include either 11 or 12 out of the 13 terms. Similiar to BMA, most of the potential covariates are included and hardly any variable selection is performed.

For SSG‐smooth, the posterior distribution of linear and nonlinear terms conditional on a given model size is shown in Figure 2 on the top right. The proportion of linear terms is shown in light grey. The proportion of nonlinear terms is split into the proportion where the corresponding linear term is also included (dark grey) or not (black). The widths of the bars are proportional to the posterior probability for models with this total number of terms. The plot indicates that even though the fitted models are larger for SSG‐smooth, not all covariates are included. This is different from SSG‐linear where nearly all covariates are always included and would have thus been identified as robust linear determinants for willingness to pay for housing. For SSG‐smooth, only a subset of the covariates is included as having either a linear and/or nonlinear effect.

Figure 2 at the bottom visualizes the combined linear and nonlinear effect of the three covariates with the highest nonlinear PIPs. In the plot the 80% confidence bands and the median of their posterior distributions of the smooth effects are provided. The identified smooth effects are similar to those given in Smith and Kohn 1996, p. 337). For the average number of rooms per dwelling (rm) a strong price increase is only detected between approximately seven and eight rooms. Regarding the percentage of lower status of the population (lstat) there is a high decrease in price if the percentage is increased at a low level. This decrease is marginally diminishing, indicating that the price decrease is smaller if the percentage is already high. With respect to the nitric oxides concentration (nox) a price decrease occurs only after a certain level is reached which seems to be at approximately 0.6.

The results for the Boston housing data indicate that some covariates might be identified as having a robust linear effect using either BMA or SSG‐linear, while their posterior model inclusion probability turns out to be negligible once all covariates are included with potentially nonlinear effects. Thus, misleading results might be obtained if only linear terms are included in the model. The identification of robust effects indeed requires more flexible models to be considered.

## 5 Application to cross‐country growth regressions

Two data sets for cross‐country growth empirics are investigated: the data set used by Sala‐i‐Martin *et al*. 2004, referred to as the SDM data set) and the data set provided by Fernández *et al*. 2001, referred to as the FLS data set). These two data sets have already been thoroughly investigated with respect to the identification of robust linear effects. The aim of the presented analyses is to uncover robust nonlinear effects on growth. The design and specification used for analysing these two data sets are the same as for the Boston housing data set with details described in section ‘Design and specifications’ and online Appendix B.3.

### The SDM data set

The data set by Sala‐i‐Martin *et al*. (2004) consists of data on 88 countries and 67 potential determinants of economic growth.^6^ Sala‐i‐Martin *et al*. (2004) used BMA techniques based on classical linear regression models to analyse this data set. They did not include any nonlinear terms.

Table 3 reports the results for BMA, SSG‐linear and SSG‐smooth. Only the results for those covariates are shown where at least one estimated PIP is larger than 0.1. Results are also shown for rather low PIPs in order to allow for a better comparison of results and assessment of the differences between the approaches, although variables with such low PIPs would not be identified as being robust determinants.

**Table 3:** SDM data set analysed using BMA, SSG‐linear and SSG‐smooth

	*BMA*			*SSG‐linear*			*SSG‐smooth*			
*Terms*	*Linear*			*Linear*			*Linear*			*Nonlin*.
*Variable*	*PIP*	*PM*	*PSC*	*PIP*	*PM*	*PSC*	*PIP*	*PM*	*PSC*	*PIP*
EAST	0.82	0.31	1.00	0.63	0.25	1.00	0.69	0.26	1.00	−
P60	0.72	0.29	1.00	0.37	0.13	0.98	0.46	0.18	0.98	0.02
IPRICE1	0.67	−0.16	0.00	0.39	−0.09	0.00	0.24	−0.05	0.01	0.02
GDPCH60L	0.60	−0.25	0.00	0.17	−0.06	0.04	0.05	−0.01	0.10	0.03
TROPICAR	0.48	−0.17	0.00	0.27	−0.08	0.02	0.31	−0.10	0.01	0.04
DENS65C	0.34	0.08	1.00	0.10	0.02	0.97	0.05	0.01	0.95	0.02
MALFAL66	0.31	−0.12	0.00	0.23	−0.07	0.03	0.14	−0.04	0.04	0.11
LIFE060	0.23	0.11	1.00	0.17	0.07	0.94	0.08	0.03	0.90	0.09
CONFUC	0.22	0.05	1.00	0.44	0.11	1.00	0.29	0.07	1.00	0.04
SAFRICA	0.17	−0.06	0.00	0.17	−0.05	0.04	0.18	−0.06	0.03	−
LAAM	0.16	−0.04	0.02	0.09	−0.02	0.06	0.06	−0.01	0.08	−
MINING	0.15	0.02	1.00	0.08	0.01	0.96	0.02	0.00	0.89	0.12
SPAIN	0.13	−0.03	0.01	0.08	−0.01	0.06	0.04	−0.01	0.11	−
MUSLIM00	0.13	0.02	0.99	0.08	0.01	0.94	0.05	0.01	0.93	0.02
GVR61	0.12	−0.02	0.00	0.12	−0.02	0.04	0.04	−0.01	0.08	0.01
BUDDHA	0.12	0.02	1.00	0.27	0.06	0.99	0.12	0.02	0.99	0.04
YRSOPEN	0.12	0.03	1.00	0.20	0.05	0.97	0.13	0.04	0.96	0.02
RERD	0.10	−0.02	0.00	0.18	−0.04	0.01	0.04	−0.01	0.07	0.01
DENS60	0.09	0.01	1.00	0.03	0.00	0.91	0.01	0.00	0.81	0.78
TROPPOP	0.07	−0.01	0.01	0.12	−0.03	0.07	0.04	−0.00	0.27	0.03
DIC	178.01			202.86			189.56			

*Notes*: nonlin: nonlinear; PIP: posterior inclusion probability; PM: posterior mean; PSC: posterior conditional positive sign certainty; DIC: deviance information criterion.

The results indicate that there are some differences between the estimated PIPs for the BMA and SSG‐linear approach for this data set. The prior parameters for the slab distribution would need to be calibrated for BMA and SSG‐linear in order to obtain a similar average posterior model size. However, even then still slight differences in the ordering of the variables according to their PIPs might occur due to the different shrinkage behaviour of the slab priors on the regression coefficients.^7^ Nonetheless, both linear approaches agree almost completely on the signs of the posterior means as indicated by the conditional positive sign certainties. The same robust positive or negative effects of the covariates on growth are identified.

SSG‐smooth only identifies one additional variable as having a robust nonlinear effect on economic growth: population density (DENS60). The nonlinear term of this covariate has a posterior inclusion probability of 0.78 and is the most frequently included term in SSG‐smooth, while its linear term has a PIP of less than 0.1 for BMA, SSG‐linear as well as SSG‐smooth. The importance of this covariate would thus have been neglected if only linear terms were included. Additional covariates which show a slight indication of a nonlinear effect are fraction GDP in mining (MINING) and malaria prevalence (MALFAL66). Both variables already have some support for inclusion as a linear term. For malaria prevalence, both the linear and the nonlinear effect have some posterior support indicating that the smooth effect estimated by SSG‐smooth contains both components, while for fraction GDP in mining the linear effect has only a very small PIP once nonlinear effects are also included.

The DIC values indicate that SSG‐smooth is slightly better fitting than SSG‐linear, while overall BMA provides the best fit. Thus, based on the model fit criterion no clear preference for the model including nonlinear terms is discerned.

**Figure 3:**
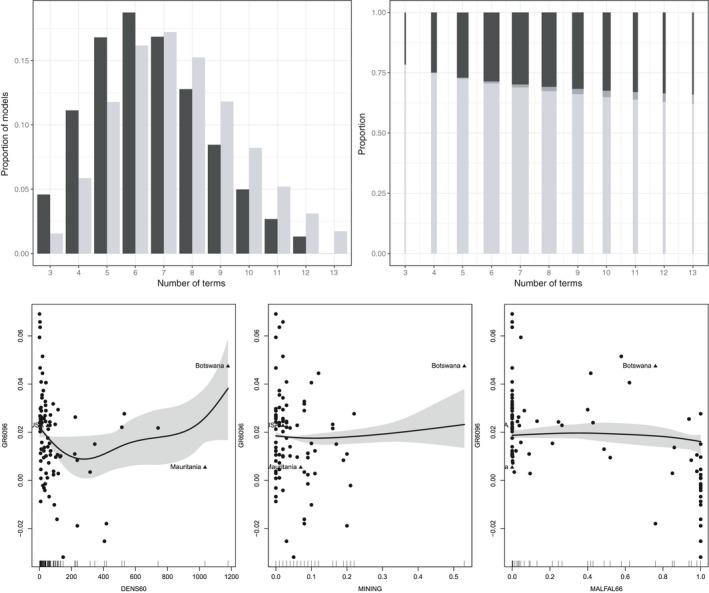
SDM data set. Top left: Distribution of model sizes for SSG‐linear (black) and SSG‐smooth (light grey). Top right: Distribution of linear terms (light grey), nonlinear terms with corresponding linear terms included (dark grey) and nonlinear terms without corresponding linear terms (black) given model size for SSG‐smooth. The bar widths are proportional to the posterior probability of the model size. Bottom: Estimated robust smooth effects indicated by the 80% confidence bands and the median of the posterior distribution.

The posterior distributions of the model sizes for SSG‐linear and SSG‐smooth are given in Figure 3 on the top left. For SSG‐linear the most frequent model size is six and the distribution is slightly right‐skewed. For SSG‐smooth the model sizes are slightly higher, with the most frequent model size being seven and the right‐skewness being more pronounced. Insights into how the models are composed of linear and nonlinear terms in the case of SSG‐smooth are provided by Figure 3 on the top right. Clearly linear terms still dominate the models. However, if nonlinear terms are included this occurs most of the time without the linear term being included. This indicates that the influence of most of the covariates does not consist of a linear effect which is in addition modulated by a nonlinear effect, but that the covariate effects are either linear or nonlinear without a linear effect (i.e., fluctuating around a horizontal line).

The smooth effects of population density (DENS60), fraction GDP in mining (MINING), and malaria prevalence (MALFAL66) are visualized in Figure 3 at the bottom. For population density, clearly the effect is decreasing for small values, while this effect flattens out for average values. The increase at the end seems to be strongly influenced by a single observation, namely Botswana. A similar effect can be observed for fraction GDP in mining. For malaria prevalence the estimated smooth effect suggests that only high values of malaria prevalence are associated with lower growth.

These visualizations indicate that SSG‐smooth is non‐robust to outliers and care has to be taken when interpreting nonlinear effects. Nonlinear effects could be identified due to outliers, which do not conform with the general linear relationship between a covariate and the dependent variable. However, estimating only linear effects would also give misleading results. The estimated linear effect would be heavily influenced by the outlier. For example, the robust linear effect of population density (DENS60) identified for BMA and SSG‐linear is positive as indicated by the PSC values which are equal to 1.00 and 0.91. However, this identified positive linear effect may be due only to the high leverage point Botswana. The visualization of the smooth effect suggests that for small values of population density there is actually a negative linear relationship between population density and economic growth.

Results for the SDM data set indicate that the population density variable has a low PIP for SSG‐linear, but turns out to be the variable with the highest PIP when nonlinear terms are also included. The estimated nonlinear effect indicates that no global linear trend is discernible for this variable because (i) the effect of the variable differs for low and high values, and (ii) there exists also a high leverage point (i.e., Botswana) which strongly influences the estimated global linear trend if only linear terms are included. The non‐robustness of linear regression thus can be alleviated by including nonlinear terms to identify outliers.

### The FLS data set

The data set by Fernández *et al*. (2001) contains cross‐country information on 72 countries and 41 potential growth determinants.^8^ Fernández *et al*. (2001) use BMA with only linear terms to identify robust determinants of growth. Their results indicate that in total 18 covariates have a PIP of at least 10% and four variables have a PIP of at least 90% (namely, GDP level in 1960 (GDP60), fraction Confucian (Confucian), life expectancy (LifeExp), equipment investment (EquipInv)).

BMA, SSG‐linear and SSG‐smooth are used to fit the FLS data set. The results are shown in Table 4. Only those covariates are included where at least for one fitted model the PIP of the linear term is larger than 0.4 or the PIP of the nonlinear term is larger than 0.1. Again results for such low PIP values are only reported to enable a better comparison of the different results.

**Table 4:** FLS data set analysed using BMA, SSG‐linear and SSG‐smooth

	*BMA*			*SSG‐linear*			*SSG‐smooth*			
*Terms*	*Linear*			*Linear*			*Linear*			*Nonlin*.
*Variable*	*PIP*	*PM*	*PSC*	*PIP*	*PM*	*PSC*	*PIP*	*PM*	*PSC*	*PIP*
GDP60	1.00	−0.79	0.00	0.73	−0.50	0.01	0.56	−0.36	0.01	0.05
Confucian	0.99	0.29	1.00	0.83	0.25	1.00	0.74	0.22	1.00	0.03
LifeExp	0.97	0.54	1.00	0.55	0.31	0.98	0.40	0.24	0.98	0.19
EquipInv	0.92	0.25	1.00	0.74	0.31	0.99	0.71	0.31	0.99	0.07
SubSahara	0.86	−0.34	0.00	0.60	−0.22	0.00	0.21	−0.07	0.02	−
Mining	0.74	0.12	1.00	0.19	0.04	0.97	0.10	0.02	0.96	0.14
RuleofLaw	0.65	0.15	1.00	0.20	0.05	0.95	0.10	0.02	0.92	0.05
NequipInv	0.63	0.10	1.00	0.18	0.03	0.96	0.18	0.04	0.98	0.19
Muslim	0.58	0.12	1.00	0.19	0.04	0.94	0.12	0.02	0.95	0.07
EcoOrg	0.58	0.09	1.00	0.17	0.03	0.97	0.11	0.02	0.97	0.03
Hindu	0.55	−0.21	0.01	0.05	−0.00	0.18	0.02	−0.00	0.25	0.07
Protestants	0.53	−0.08	0.00	0.28	−0.06	0.02	0.16	−0.03	0.02	0.06
LabForce	0.48	0.17	0.97	0.04	0.00	0.57	0.02	0.00	0.57	0.04
LatAmerica	0.47	−0.12	0.03	0.26	−0.06	0.04	0.20	−0.05	0.03	−
BlMktPm	0.47	−0.05	0.00	0.06	−0.01	0.13	0.03	−0.00	0.23	0.10
EthnoL	0.41	0.08	0.99	0.05	0.00	0.66	0.03	0.00	0.69	0.04
YrsOpen	0.36	0.08	0.94	0.41	0.12	0.98	0.48	0.17	0.99	0.03
Catholic	0.22	−0.01	0.39	0.09	−0.01	0.18	0.05	−0.01	0.15	0.17
DIC	99.22			172.86			167.03			

*Notes*: nonlin: nonlinear; PIP: posterior inclusion probability; PM: posterior mean; PSC: posterior conditional positive sign certainty; DIC: deviance information criterion.

PIPs are clearly higher for BMA than for SSG‐linear. To obtain a similar average posterior model size for both approaches the prior setting for the slab distributions would need to be calibrated. However, even if proportionally adjusting for the mean posterior model size some differences may remain for the PIPs obtained using the two approaches. This might be due to the different shrinkage behaviour of the two priors imposed on the regression coefficients. SSG adaptively shrinks each coefficient differently and also induces a stronger shrinkage on variables with large contribution to low‐variance principal components (see online Appendix B.4). Furthermore, Hofmarcher *et al*. (2014) indicate that even if the same prior structure is used for the regression coefficients in a BMA analysis, the relative importance assigned to variables might change for increasing model size. They indicate that a variable which tends to be included for small model sizes might have a decreasing posterior weight for larger model sizes because several other variables are included for the larger model sizes essentially capturing the influence of this variable in a better way and replacing its inclusion.

Comparing SSG‐linear and SSG‐smooth indicates that most of the top ten variables identified for SSG‐linear are also frequently included for SSG‐smooth. Furthermore, for SSG‐smooth the highest PIPs are observed only for linear terms. The nonlinear effect with the highest PIP is observed for non‐equipment investment (NequipInv) and this variable has rank nine when effects are sorted by PIP. Furthermore, the DIC values reported are quite similar for SSG‐linear and SSG‐smooth indicating a slight preference for SSG‐smooth. The DIC value for BMA is clearly smaller than for SSG‐linear and SSG‐smooth suggesting that a model including nonlinear terms is not required to achieve a good fit, but that BMA is preferable regarding model fit.

**Figure 4:**
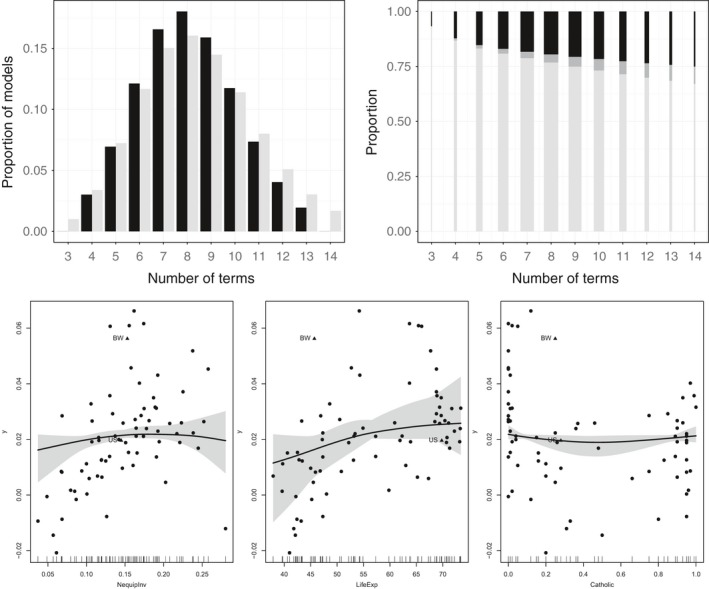
FLS data set. Top left: Distribution of model sizes for SSG‐linear (black) and SSG‐smooth (light grey). Top right: Distribution of linear terms (light grey), nonlinear terms with corresponding linear terms included (dark grey) and nonlinear terms without corresponding linear terms (black) given model size for SSG‐smooth. The bar widths are proportional to the posterior probability of the model size. Bottom: Estimated robust smooth effects indicated by the 80% confidence bands and the median of the posterior distribution.

SSG‐linear and SSG‐smooth lead to rather similar posterior average model sizes (8.2 and 8.4). The posterior model size distributions for the two SSG approaches are shown in Figure 4 on the top left. For both the most frequent model size is eight. In Figure 4 on the top right it can be seen – similar to the SDM data set – that linear terms are included most often while nonlinear terms are only included if the corresponding linear term is not part of the model.

The smooth effects most frequently included in the model are visualized in Figure 4 at the bottom with their 80% confidence bands and the median of their posterior distributions. The smooth effects identified for non‐equipment investment (NequipInv) and fraction Catholic (Catholic) provide a slight indication that extreme values for these covariates have a different effect than an average value. For life expectancy (LifeExp) the estimated robust smooth effect slightly indicates that the influence of the covariate shows diminishing returns for higher values.

For the FLS data set SSG‐smooth validates and confirms the results obtained with SSG‐linear and BMA. The results indicate that more complex functional relationships are not required to identify robust determinants and similar linear effects are discerned if the assumption of a constant linear effect is relaxed.

The results for the two growth data sets indicate that BMA might already be suitable for these data sets and the additional complexity of SSG is not required. Only slight nonlinear effects between the covariates and the dependent variable are detected or are even only identified because of outlying observations. Effects of outlying observations could also be captured within a standard BMA setting by either removing these observations or including dummy variables as covariates. However, these insights are only available if the more complex model is evaluated. Only then can the simpler model be safely selected and interpreted.

## 6 Conclusions

In this paper the usefulness of SSG proposed by Scheipl *et al*. (2012) is indicated for economic regression analysis under variable and functional relationship uncertainty. SSG is a powerful tool to address two types of model uncertainty simultaneously: uncertainty about the explaining variables (variable uncertainty) and uncertainty about the functional form of the variables’ impact (linear vs. nonlinear effects). SSG enables researchers to identify the covariates with (i) robust linear effects and (ii) robust nonlinear effects on the outcome variable. The unknown nonlinear effects are modelled based on a flexible penalized spline approach. Relevant variables along with their functional relationships are identified based on stochastic search variable selection techniques.

The approach has several benefits: (i) misleading results are avoided, which might be obtained if nonlinear functional relationships between covariates and the dependent variable are neglected, (ii) results obtained using only linear terms can be validated by verifying that adding nonlinear effects does not change the identification of the robust linear effects, (iii) covariates without a linear, but only with a nonlinear effect, are not disregarded as important determinants for the dependent variable, (iv) differences in marginal effects can be identified which might have important policy implications, and (v) postprocessing tools available for BMA can be reused. SSG can also be employed in an exploratory way to identify nonlinear terms to be selectively included in a standard BMA application.

Finally, we want to emphasize that SSG‐smooth can be easily applied using software available for the R environment for statistical computing and graphics (R Core Team, 2018) and implemented in the package spikeSlabGAM (Scheipl, 2011). The results presented in this paper are obtained using the default setting for the prior parameters in the package. Thus, no extensive tuning of the hyper‐parameters of the priors is required in order to arrive at useful solutions. Overall this suggests that any empirical economics analysis based on regression models and facing variable and functional relationship uncertainty should not rely on the assumption of ‘knowing’ a priori the functional relationship or imposing a simplified linear relationship. In contrast, robust determinants should be identified using suitable methods which account for variable and functional relationship uncertainty.

## Supporting information


**Appendix B.** Methodological details.

## Notes

1 For more details and properties of the peNMIG prior as well as the hierarchical representation including an indicator variable *γ* indicating assignment to the slab see online Appendix B. In the online appendix also a detailed comparison between BMA and SSG is given.

2 The derivation of the DIC for BMA where due to collapsed Gibbs sampling no MCMC samples are available for all parameters is given in online Appendix B.5.

3 More details on the software used and hyper‐parameter values specified are given in online Appendix B.3.

4 More details on the specified prior parameters are given in online Appendix B.3.

5 All variables are summarized with their names and a description in Table A1 in Appendix A.

6 All variables are summarized with their names and a description in Table A2 in Appendix A.

7 See the discussion in online Appendix B.4 on the difference between BMA and SSG.

8 All variables are summarized with their names and a description in Table A3 in Appendix A.

## Appendix 1 A

Tables A1, A2, A3

**Table A1:** Variables and their description for the Boston housing data set

*Variable name*	*Description*
crim	*Per capita* crime rate by town
zn	Proportion of residential land zoned for lots over 25,000 sq.ft
indus	Proportion of non‐retail business acres per town
chas	Charles River dummy variable (= 1 if tract bounds river; 0 otherwise)
nox	Nitric oxides concentration (parts per 10 million)
rm	Average number of rooms per dwelling
age	Proportion of owner‐occupied units built prior to 1940
dis	Weighted distances to five Boston employment centres
rad	Index of accessibility to radial highways
tax	Full‐value property‐tax rate per USD 10,000
ptratio	Pupil‐teacher ratio by town
b	1000(*B*−0.63)^2^ where *B* is the proportion of blacks by town
lstat	Percentage of lower status of the population
medv	Median value of owner‐occupied homes in USD 1000's

**Table A2:** Variables and their description for the SDM data set

*Variable name*	*Description*
ABSLATIT	Absolute latitude
AIRDIST	Air distance to big cities
AVELF	Ethnolinguistic fractionalization
BRIT	British colony
BUDDHA	Fraction Buddhist
CATH00	Fraction Catholic
CIV72	Civil liberties
COLONY	Colony dummy
CONFUC	Fraction Confucian
DENS60	Population density coastal 1960's
DENS65C	Population density 1960
DENS65I	Interior density
DPOP6090	Population growth rate 1960–90
EAST	East Asian dummy
ECORG	Capitalism
ENGFRAC	English‐speaking population
EUROPE	European dummy
FERTLDC1	Fertility in 1960's
GDE1	Defence spending share
GDPCH60L	GDP 1960 (log)
GEEREC1	Public education spending share in GDP in 1960's
GGCFD3	Government consumption share deflated with GDP prices
GOVNOM1	Nominal government GDP share 1960's
GOVSH61	Government share of GDP
GR6096	Average growth rate of GDP *per capita* 1960–96
GVR61	Government consumption share 1960's
H60	Higher education in 1960
HERF00	Religious intensity
HINDU00	Fraction Hindu
IPRICE1	Investment price
LAAM	Latin American dummy
LANDAREA	Land area
LANDLOCK	Landlocked country dummy
LHCPC	Hydrocarbon deposits in 1993
LIFE060	Life expectancy in 1960
LT100CR	Fraction of land area near navigable water
MALFAL66	Malaria prevalence in 1960's
MINING	Fraction GDP in mining
MUSLIM00	Fraction Muslim
NEWSTATE	Time of independence
OIL	Oil‐producing country dummy
OPENDEC1	(Imports + exports)/GDP
ORTH00	Fraction Orthodox
OTHFRAC	Fraction speaking foreign language
P60	Primary schooling 1960
PI6090	Average inflation 1960–90
POP1560	Fraction population less than 15
POP60	Population in 1960
POP6560	Fraction population over 65
PRIEXP70	Primary exports in 1970
PRIGHTS	Political rights
PROT00	Fraction Protestant
RERD	Real exchange rate distortions
REVCOUP	Revolution and coups
SAFRICA	African dummy
SCOUT	Outward orientation
SIZE60	Size of the economy
SOCIALIST	Socialist dummy
SPAIN	Spanish colony
SQPI6090	Square of inflation 1960–90
TOT1DEC1	Terms of trade growth in 1960's
TOTIND	Terms of trade ranking
TROPICAR	Fraction of tropical area
TROPPOP	Fraction population in tropics
WARTIME	Fraction spent in war 1960–90
WARTORN	War participation 1960–90
YRSOPEN	Years open
ZTROPICS	Tropical climate zone

**Table A3:** Variables and their description for the FLS data set

*Variable name*	*Description*
y	Economic growth 1960–92 as from the Penn World Tables Rev 6.0
Abslat	Absolute latitude
Spanish	Spanish colony dummy
French	French colony dummy
Brit	British colony dummy
WarDummy	War dummy
LatAmerica	Latin America dummy
SubSahara	Sub‐Sahara dummy
OutwarOr	Outward orientation
Area	Area surface
PrScEnroll	Primary school enrolment
LifeExp	Life expectancy
GDP60	Initial GDP in 1960
Mining	Fraction of GDP in mining
EcoOrg	Degree of capitalism
YrsOpen	Number of years having an open economy
Age	Age
Buddha	Fraction Buddhist
Catholic	Fraction Catholic
Confucian	Fraction Confucian
EthnoL	Ethnolinguistic fractionalization
Hindu	Fraction Hindu
Jewish	Fraction Jewish
Muslim	Fraction Muslim
PrExports	Primary exports 1970
Protestants	Fraction Protestants
RuleofLaw	Rule of law
Popg	Population growth
WorkPop	Workers per inhabitant
LabForce	Size of labour force
HighEnroll	Higher education enrolment
PublEdupct	Public education share
RevnCoup	Revolutions and coups
PolRights	Political rights
CivlLib	Civil liberties
English	Fraction speaking English
Foreign	Fraction speaking foreign language
RFEXDist	Exchange rate distortions
EquipInv	Equipment investment
NequipInv	Non‐equipment investment
stdBMP	Stand. dev. of black market premium
BlMktPm	Black market premium
