# Supplementary material for: Semi‐parametric Regression under Model Uncertainty: Economic Applications
Source: Oxf Bull Econ Stat. 2019 Feb 19;81(5):1117–43. doi: 10.1111/obes.12294 (PMC6850473; doi:10.1111/obes.12294)
Supplement: Supplementary file 1 — Appendix B. Methodological details. [file OBES-81-1117-s001.pdf]

B Methodological details

B.1 NMIG prior

In order to perform variable selection in regression models, Ishwaran and Rao (2005) proposed the normal-mixture of inverse Gamma (NMIG) prior for the linear regression coefficients:

$$\beta_k \sim \text{NMIG}(v_0, w, a_\tau, b_\tau), \quad k = 1, \dots, K,$$

with an additional prior on the sizes of the two-component mixture $(w, 1-w)$ given by

$$w \sim \text{Beta}(a, b). \tag{B.1}$$

The NMIG prior can be defined using a hierarchical representation where latent allocation variables γ_k are introduced for the mixture to indicate if the coefficients are assigned to the spike or the slab:

$$\begin{aligned}\beta_k|\gamma_k, \tau_k^2 &\sim N(0, ((1-v_0)\gamma_k + v_0)\tau_k^2), \quad k = 1, \dots, K,\\ \gamma_k &\sim \text{Bernoulli}(w), \quad k = 1, \dots, K,\\ \tau_k^2 &\sim \text{Gamma}^{-1}(a_\tau, b_\tau), \quad k = 1, \dots, K.\end{aligned}$$

The NMIG prior uses a non-degenerate spike distribution which is a re-scaled version of the slab distribution, i.e., the slab variance is multiplied by a small fixed constant v_0. For the slab a hierarchical prior distribution is used consisting of a normal distribution with variance τ_k^2 and an inverse Gamma distribution for the variance τ_k^2.

B.2 peNMIG prior

Scheipl *et al.* (2012) propose the parameter expanded NMIG (peNMIG) prior as an extension to the NMIG prior which is particularly suitable to simultaneously assign all coefficients $\boldsymbol{\delta}_k$ corresponding to the non-linear term of variable k jointly to the spike or slab component. The peNMIG prior is based on a multiplicative parameter expansion strategy which introduces only partially identifiable working parameters to enable simultaneous selection or deselection of large coefficient batches. In this way a good mixing behaviour of the MCMC sampler is retained. This would not be achieved if the NMIG prior were directly used as a multivariate prior for simultaneous inclusion and exclusion of a set of coefficient values. In the peNMIG prior a scalar parameter is used to decide if all coefficients $\boldsymbol{\delta}_k$ corresponding to the same linear or non-linear effect are assigned to the spike or slab.

Online appendix: doi:10.1111/obes.12294.

In SSG the peNMIG prior is specified for both sets of regression coefficients:

$$\beta_k \sim \text{peNMIG}(v_0, w, a_\tau, b_\tau), \quad k = 1, \dots, K,$$
$$\boldsymbol{\delta}_k \sim \text{peNMIG}(v_0, w, a_\tau, b_\tau), \quad k = 1, \dots, K,$$

with w drawn as for the NMIG prior given in Equation (B.1).

The hierarchical representation of the peNMIG prior for $\boldsymbol{\delta}_k$ is given by

$$\begin{aligned}
\boldsymbol{\delta}_k &= \zeta_k \boldsymbol{\xi}_k, \quad k = 1, \dots, K,\\
\zeta_k &\sim N(0, ((1 - v_0)\gamma_k^{\text{smooth}} + v_0)\tau_k^2), \quad k = 1, \dots, K,\\
\xi_{lk} &\sim N(m_{lk}, 1), \quad l = 1, \dots, L_k; k = 1, \dots, K,\\
m_{lk} &\sim \frac{1}{2}\delta_1(m_{lk}) + \frac{1}{2}\delta_{-1}(m_{li}), \quad l = 1, \dots, L_k; k = 1, \dots, K,\\
\gamma_k^{\text{smooth}} &\sim \text{Bernoulli}(w), \quad k = 1, \dots, K,\\
\tau_k^2 &\sim \text{Gamma}^{-1}(a_\tau, b_\tau), \quad k = 1, \dots, K,
\end{aligned}$$

The univariate variable ζ_k is used for updating γ_k^{smooth} and τ_k^2. The vector $\boldsymbol{\xi}_k$ has the absolute values of its entries shrunken towards one through the mixture prior with means at ± 1 in order to ensure that in fact ζ_k captures the "importance" of $\boldsymbol{\delta}_k$. The hierarchical representation of the peNMIG prior for β_k is analogous to the one of $\boldsymbol{\delta}_k$ with γ_k^{lin} representing the inclusion indicator for the linear term of the kth covariate.

The peNMIG prior shows desirable shrinkage properties if the marginal priors on the regression coefficients are analysed. The marginal peNMIG prior has a spike at zero and heavy tails which imply redescending score functions. This ensures Bayesian robustness of the resulting shrinkage estimators. This desirable combination is similar to other shrinkage priors, including the horseshoe prior (Carvalho *et al.* 2009). A detailed analysis of the shrinkage properties is given in Scheipl *et al.* (2012). In addition the marginal prior has been shown to be rather insensitive to the specific choice of the prior parameter values v_0, a_τ and b_τ.

B.3 Inference and hyper-parameter values

For model fitting the R package BMS (Zeugner and Feldkircher 2015) is used for BMA and the R package spikeSlabGAM (Scheipl 2011) for SSG. SSG is used to fit two different model classes: (1) a model class including only linear terms ("SSG-linear") and (2) a model class including linear and non-linear terms ("SSG-smooth").

For both packages the default settings for the prior specifications are used. That is, for BMA the hyper-parameters are set to $a = b = 1$ and $g = n$ in the g-prior for the regression coefficients. This implies that a uniform prior is used for the prior inclusion probabilities of the

single covariates and that the unit information prior is used for the g-prior as recommended by Eicher *et al.* (2011). In addition an improper prior corresponding to the Jeffreys prior is used for σ^2.

For SSG the hyper-parameters for w are also set to $a = b = 1$. Thus the same prior setting is used for the variable inclusion parameter for both approaches. In addition the scaling factor for the spike is set to $v_0 = 0.00025$ and the hyper-parameters for τ_k^2 to $a_\tau = 5$, $b_\tau = 25$. Scheipl (2011) suggests these values based on validations made in many simulations and data examples. The shrinkage properties of the peNMIG prior have been shown to be fairly robust to the specific choice of v_0, a_τ, and b_τ (Scheipl *et al.* 2012). Spline basis expansions for the non-linear terms are used with 20 cubic B-spline basis functions with equidistant knots across the covariates' ranges in the original basis. Non-linear terms are only included for variables with at least 3 unique different values, i.e., binary variables are excluded. The hyper-parameters for σ^2 are set to $a_\sigma = b_\sigma = 0.0001$ implying that a proper prior distribution is used for σ^2 which approximates $p(\sigma^2) \propto \sigma^2$ (Lunn *et al.* 2012, p. 87). Hence the prior for σ^2 is close to the improper prior used in BMA.

B.4 A joint framework for BMA and SSG-linear

In the following a prior specification for the regression coefficients $\boldsymbol{\beta} = (\beta_1, \ldots, \beta_K)$ and the other parameters is considered which represents a general framework encompassing BMA and SSG as special cases:

$$\begin{aligned}
\boldsymbol{\beta} &\sim N\left(\mathbf{0}, \text{diag}(\boldsymbol{\nu})\boldsymbol{A}\text{diag}(\boldsymbol{\nu})\right), \\
\nu_k &= \sqrt{((1 - v_0)\gamma_k + v_0\tau_k}, \quad k = 1, \ldots, K, \\
\gamma_k &\sim \text{Bernoulli}(w), \quad k = 1, \ldots, K, \\
w &\sim \text{Beta}(a, b), \\
\tau_k^2 &\sim \text{Gamma}^{-1}(a_\tau, b_\tau), \quad k = 1, \ldots, K, \\
\alpha, \sigma^2 &\propto \text{Gamma}^{-1}(a_\sigma, b_\sigma).
\end{aligned}$$

where $\boldsymbol{\nu} = (\nu_1, \ldots, \nu_K)$ and diag$(\cdot)$ creates a diagonal matrix.

In BMA the following specifications are used:

$$\boldsymbol{A} = \left(\frac{1}{g}\boldsymbol{X}^\top\boldsymbol{X}\right)^{-1}, \qquad v_0 = 0, \qquad g = n, \qquad \tau_k^2 \equiv \tau^2 = \sigma^2 \propto \frac{1}{\sigma^2}$$

for all $k = 1, \ldots, K$. The variance of the regression coefficients τ_k^2 is assumed to be equal for all $k = 1, \ldots, K$ and coupled with the variance of the noise σ^2. In addition a fixed value for g corresponding to the unit information prior is used.

In SSG the specifications used are:

$$\boldsymbol{A} = \boldsymbol{I}, \qquad v_0 = 0.00025, \qquad a_\tau = 5, b_\tau = 25, \qquad a_\sigma = b_\sigma = 0.0001.$$

where $\boldsymbol{I}$ is the identity matrix of dimension K.

The following differences between BMA and SSG can be observed:

(1) The spike distribution corresponds to a degenerate Dirac distribution in BMA, whereas SSG uses a continuous non-degenerate distribution. This corresponds to a different choice of the parameter v_0 which is set to zero for BMA and equal to 0.00025 in SSG. As a consequence, regressors where coefficients are assigned to the spike, are actually excluded from the model in BMA, whereas in SSG they are always included in the model. Coefficients assigned to the spike have values which are different from zero, but are very small.

(2) The slab distributions are g-priors in BMA. In BMA the variance of the slab distribution is scaled by the residual variance and the parameter g. The value of g in BMA influences the posterior inclusion probabilities and thus the posterior model sizes. In SSG the variance is scaled by a separate parameter specific to each linear or non-linear term. These parameters are adaptively determined in a data-driven way by imposing a hyper-prior on them. This adaptivity is important to select a suitable smoothness for the non-linear terms. It also increases the complexity of the model fitted with SSG compared to BMA. In SSG for each linear and non-linear term a suitable shrinkage value is adaptively determined. In BMA the same amount of shrinkage is applied to all regression coefficients using the fixed parameter g.

(3) The different values for $\boldsymbol{A}$ induce a different shrinkage behaviour. In case of BMA each OLS regression coefficient is shrunken proportionally, whereas in SSG the OLS regression coefficients are shrunken along the principal components with coefficients contributing to low-variance principal components being shrunken more.

(4) An improper prior on σ^2 is used in BMA. For SSG an inverse Gamma prior is used with very small values (i.e., 0.0001) for the hyper-parameters inducing a proper prior. The inverse Gamma prior used in SSG converges to the improper prior of BMA if both hyper-parameter values go to zero.

In BMA and SSG the same prior structure is employed for the variable/term inclusion indicators $\boldsymbol{\gamma}$. Despite the matching of the prior inclusion probabilities, differences in the posterior inclusion probabilities might occur due to the lack of matching of the slab distributions as well as their different shrinkage behaviour. A careful matching of the slab priors would be required to at least induce the same mean posterior model size.

BMA has the advantage that the marginal likelihoods are analytically given. Thus, the indicator vector for variable inclusion $\boldsymbol{\gamma}$ can be directly sampled without conditioning on the regression coefficients using a collapsed Gibbs sampler. The posterior distributions for the regression coefficients are determined in a separate step and can be calculated in closed form given the posterior distribution of $\boldsymbol{\gamma}$. In SSG an expanded Gibbs sampling scheme is employed where the indicator vector for variable inclusion $\boldsymbol{\gamma}$ is sampled along with the regression coefficients and the penalty parameters which have hierarchical priors.

B.5 DIC calculation for BMA

The DIC is given by

$$\text{DIC} = \overline{D(\boldsymbol{\theta})} + p_D = 2\overline{D(\boldsymbol{\theta})} - D(\overline{\boldsymbol{\theta}}),$$

where $D(\cdot)$ is the deviance and $\overline{D(\boldsymbol{\theta})}$ is the mean posterior deviance, while $D(\overline{\boldsymbol{\theta}})$ is the deviance at the posterior mean of the full parameter vector $\boldsymbol{\theta}$. The DIC can easily be calculated if posterior draws from the full parameter vector are available.

For BMA collapsed Gibbs sampling is performed for posterior inference. Thus no posterior draws of the regression coefficients and the error variance are available. In this case the DIC value can be obtained for BMA in the following way. According to Spiegelhalter *et al.* (2002) the effective number of parameters p_D of a single linear model are given by

$$p_D \approx \frac{g}{1+g}K + 2 + \frac{1}{3n},$$

where K denotes the number of covariates in the linear model (without the intercept). The mean posterior deviance of a single linear model is equal to

$$\overline{D(\theta)} = n\log(2\pi) + p_D + \bar{\kappa}(\boldsymbol{y} - \boldsymbol{X}\bar{\beta})^\top(\boldsymbol{y} - \boldsymbol{X}\bar{\beta}) - n\log(\bar{\kappa})$$

with

$$\bar{\kappa} = n\left(\boldsymbol{y}^\top\boldsymbol{y} - \frac{1+g}{g}\bar{\beta}^\top\boldsymbol{X}^\top\boldsymbol{X}\bar{\beta}\right)^{-1},$$

$\bar{\beta}$ the posterior mean estimate of the regression coefficients and $\overline{\sigma^2} = \frac{1}{\bar{\kappa}}\frac{n}{n-1}$. The DIC for BMA is then calculated by using the weighted mean of the average deviances for each linear model to obtain the posterior mean deviance. For the deviance at the posterior mean the posterior mean estimates are determined by the weighted means of the posterior mean coefficients and of the posterior mean error variance estimates of the single linear models.

References

Carvalho CM, Polson NG, Scott JG (2009). "Handling Sparsity via the Horseshoe." In *Proceedings of the 12th International Conference on Artificial Intelligence and Statistics (AISTATS)*, pp. 73–80. Clearwater Beach, Florida, USA.

Eicher TS, Papageorgiou C, Raftery AE (2011). "Default Priors and Predictive Performance In Bayesian Model Averaging, with Application To Growth Determinants." *Journal of Applied Econometrics*, **26**(1), 30–55.

Ishwaran H, Rao JS (2005). "Spike and Slab Variable Selection: Frequentist and Bayesian Strategies." *The Annals of Statistics*, **33**(2), 730–773.

Lunn D, Jackson C, Best N, Thomas A, Spiegelhalter D (2012). *The BUGS Book – A Practical Introduction to Bayesian Analysis.* CRC Press / Chapman and Hall.

Scheipl F (2011). "spikeSlabGAM: Bayesian Variable Selection, Model Choice and Regularization for Generalized Additive Mixed Models in R." *Journal of Statistical Software*, **43**(14), 1–24.

Scheipl F, Fahrmeir L, Kneib T (2012). "Spike-and-Slab Priors for Function Selection In Structured Additive Regression Models." *Journal of the American Statistical Association*, **107**(500), 1518–1532.

Spiegelhalter DJ, Best NG, Carlin BP, van der Linde A (2002). "Bayesian Measures of Model Complexity and Fit." *Journal of the Royal Statistical Society, Series B*, **64**(4), 583–639.

Zeugner S, Feldkircher M (2015). "Bayesian Model Averaging Employing Fixed and Flexible Priors: The BMS Package for R." *Journal of Statistical Software*, **68**(4), 1–37.
